# Microscopic colitis is associated with an increased risk of dementia in a Swedish population

**DOI:** 10.1111/joim.70046

**Published:** 2025-11-26

**Authors:** Xiaoying Kang, David Bergman, Jiangwei Sun, Karin Wirdefeldt, Jonas F. Ludvigsson

**Affiliations:** ^1^ Department of Medical Epidemiology and Biostatistics Karolinska Institutet Solna Sweden; ^2^ Department of Neurology Brigham and Women's Hospital & Harvard Medical School Boston Massachusetts USA; ^3^ Department of Neurology Yale School of Medicine New Haven Connecticut USA; ^4^ Department of Clinical Neuroscience Karolinska Institutet Solna Sweden; ^5^ Department of Pediatrics Örebro University Hospital Örebro Sweden; ^6^ Department of Medicine Columbia University College of Physicians and Surgeons New York New York USA

**Keywords:** Alzheimer's disease, Dementia, Microscopic colitis, Matched cohort study, Matched case‐control studys

## Abstract

**Background:**

The microbiota–gut–brain axis has been implicated in dementia. Yet whether dementia is associated with microscopic colitis (MC), an age‐related inflammatory colonic disease involving gut dysbiosis, remains unknown.

**Methods:**

Using the nationwide ESPRESSO cohort in Sweden, we compared MC patients histologically diagnosed 1990–2017 and aged ≥30 years to their population‐based comparators and siblings, separately. MC association with incident and prevalent dementia diagnosis, respectively, was investigated in a matched cohort and a matched case‐control design.

**Findings:**

Following 13,037 MC patients and 61,710 population comparators for a median of ∼10 years, we observed 4674 incident dementia cases (46% were Alzheimer's disease [AD]). During the first 5 years since biopsy, MC was associated with a 19% higher dementia risk (adjusted hazard ratio [aHR]: 1.19; 95% confidence interval [CI]: 1.07–1.32). This short‐term association applied to both AD and vascular dementia and appeared stronger as compared to siblings (aHR: 1.55; 95% CI: 1.22–1.97). After 5 years, it attenuated to null in both comparisons, regardless of dementia subtype. Prior dementia was less prevalent in MC (adjusted odds ratio [aOR]: 0.73; 95% CI: 0.65–0.82). This inverse association was independent from medications commonly prescribed in MC but was not supported by sibling findings (aOR: 1.11; 95% CI: 0.81–1.51).

**Conclusions:**

MC patients may be more vulnerable to dementia diagnosis in early disease course. The intriguing inverse association between MC and preexisting dementia implies a possible underdiagnosis of MC in demented population and warrants further investigation.

## Graphical abstract

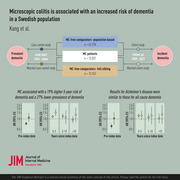

**Abbreviations**
AD: Alzheimer's disease
ATC: Anatomical Therapeutic Chemical
BMI: body mass index
CC: collagenous colitis
CDR: cause of Death Register
CI: confidence interval
ESPRESSO: Epidemiology Strengthened by histopathology Reports in Sweden
HR: hazard ratio
IBD: inflammatory bowel disease
ICD: International Classification of Diseases
LC: lymphocytic colitis
MC: microscopic colitis
NPR: National Patient Register
OR: odds ratio
PDR: Prescribed Drug Register
PPI: proton pump inhibitors
SSRI: selective serotonin reuptake inhibitor
VaD: vascular dementia

## Introduction

Microscopic colitis (MC) is an inflammatory condition of the large intestine [1]. The disease is usually characterized as either collagenous colitis (CC) or lymphocytic colitis (LC), distinguished by their respective histopathological presentation [1]. Clinically, MC presents with watery and non‐bloody diarrhea, although other symptoms like weight loss, abdominal pain, urgency, and fecal incontinence can occur [2]. Macroscopically, there are no pathognomonic findings, and the mucosa often appears normal or nearly normal with unspecific inflammatory changes [1]. Diagnosis of MC thus requires both colonoscopy and properly assessed biopsies from the colon. Previous studies have found MC to be a disease of the elderly, with a median age of disease onset at ∼60 years. Incidence rates of MC have increased markedly over the past decades [3], now rivaling those of classical inflammatory bowel diseases (IBD) [4]. Although IBD has been linked to dementia [5], suggesting the role of chronic inflammation [6] and intestinal dysbiosis acting along the microbiota–gut–brain axis [7], little is known about the potential relation between MC and dementia and relevant literature is limited to a single case‐report [8].

Similar to IBD, MC has been associated with intestinal dysbiosis [9], which can disrupt the gut–brain communication via microbial metabolites, inflammatory mediators, and increased gut permeability [7]. Furthermore, several key inflammatory pathways involved in dementia‐related neuroinflammation [7], including the elevation of pro‐inflammatory cytokines such as tumor necrosis factor‐alpha, interleukin‐6, and interleukin‐22 [1], have also been implicated in MC. In light of these overlapping inflammatory mechanisms shared by MC with IBD [10] and dementia [7], we hypothesize that MC patients may be at increased risk of developing dementia or having an earlier dementia onset and advocate for research on potential neurological implications of MC.

In the present study, we sought to characterize comprehensively the relationship between MC and dementia using nationwide register data in Sweden.

## Methods

### Study design

MC association with subsequent and prior diagnosis of dementia was examined in a matched cohort (1990–2021) and a matched case‐control design, respectively, using the nationwide ESPRESSO cohort as study base [11].

### Data sources

Data from several Swedish national registers were analyzed. The ESPRESSO study collected histopathology reports from the gastrointestinal tract between 1965 and 2017 from all 28 pathology departments in Sweden [11]. In ESPRESSO, each person with a biopsy is defined as an “index patient” and the date of biopsy is used as “index date.” We used two comparison groups from ESPRESSO: (1) “population comparators,” which consist of up to five individuals without any biopsy indicative of the index disease matched from the general population for each index patient on age, sex, calendar year, and county of residence at index year and (2) “sibling comparators,” which include all full siblings of each index patient who had no biopsy indicative of the index disease. Demographics, such as date and country of birth, death, educational attainment, and migration, of index patients and their population and sibling comparators were extracted from the Total Population Register [12] and the longitudinal integrated database for health insurance and labor market studies [13]. Data on disease diagnosis, such as date of admission and discharge and type of diagnosis (primary or secondary), was queried from two sources via data linkage at individual level: (1) the National Patient Register (NPR), which contains records of hospital visits to inpatient (available since 1964 and reached 100% coverage since 1987) and outpatient care (available since 2001) but not primary care and (2) the Cause of Death Register (CDR), which contains the underlying and contributing causes of death since 1961 [14]. In NPR, the Swedish‐version International Classification of Diseases (ICD) was used to code diagnoses; whereas in CDR, the standard ICD classification was used to code diagnoses. Established in July 2005, the Prescribed Drug Register (PDR) provides virtually complete information about prescription drugs dispensed at pharmacies based on the Anatomical Therapeutic Chemical (ATC) code [15].

### Ascertainment of MC patients and their comparators

MC was ascertained from the ESPRESSO cohort based on two biopsy classification codes: “M40600” for CC and “M47170” for LC. According to our earlier validation study, such biopsy‐confirmed MC diagnosis is 95% concordant to the clinical diagnosis of MC [2]. Date of MC diagnosis was then defined as the date of the MC‐indicating biopsy. Given its rare diagnosis in Sweden before 1990 [3], we included only MC patients diagnosed 1990–2017. For each included index MC patient, we extracted their matched population comparators and MC‐free siblings. Of note, for subjects who were initially MC‐free and matched to an index patient and who received MC diagnosis later on, we censored them as comparators at the time of the recorded MC diagnosis, re‐matched them to their own set of MC‐free comparators at that index year, and followed them up as index patients since then. Next, two exclusion criteria were applied: First, we excluded those aged <30 years at index date as both MC and dementia are age‐related diseases; second, we excluded those with immigration or emigration records within ±3 years around index date to minimize misclassification due to delayed registration or de‐registration [16, 17].

### Ascertainment of dementia and its subtypes

Diagnosis of all‐cause dementia was ascertained as previously described [18, 19, 20]. Briefly, each dementia subtype, namely, Alzheimer's disease (AD), vascular dementia (VaD), or other dementia, was identified from the NPR and CDR based on the ICD codes listed in Table S1, considering both primary and secondary diagnosis and both underlying and contributing cause of death. For AD specifically, we also extracted the history of prescribing anti‐dementia drugs from the PDR based on the ATC codes (Table S1). For each subtype of dementia, date of diagnosis was defined per Eriksson et al. [20] as the earliest among (1) 3 years before the first NPR‐based diagnosis, (2) 5 years before the first CDR‐based diagnosis, and (3) the first PDR‐based diagnosis. All‐cause dementia was then defined for patients diagnosed with any dementia subtype. When a patient was diagnosed with only one dementia subtype, date of being diagnosed for that subtype was used as date of all‐cause dementia; otherwise, date of all‐cause dementia was based on the subtype diagnosed first.

### Covariates

Three matching variables, namely, index age, sex, and index year, as well as educational attainment (four categories per years of education: ≤9, 10–12, ≥13, and missing), were adjusted for in all analyses. For population analysis, birth country was also included as a binary variable indicating whether born in Nordic countries (Sweden, Norway, Denmark, Finland, or Iceland) or not. Throughout the analysis, five comorbidities—chronic obstructive pulmonary disease (as a proxy for heavy smoking), IBD, type 1 diabetes, unipolar depression, and anxiety—were accounted for by including a dummy variable indicating whether an individual had any of these conditions. These comorbidities were adjusted for as confounding variable due to their associations with both MC and dementia and their low likelihood of being a mediator of the potential MC‐dementia relationship [1, 21, 22, 23, 24, 25, 26, 27, 28, 29, 30]. To minimize confounding by healthcare‐seeking behaviors, number of inpatient and outpatient visits occurred between 3 years and 6 months prior to index date were counted and dichotomized at its median value for statistical adjustment. In a sensitivity analysis (see Statistical Analysis), pre‐index use of three drugs commonly related to MC onset, namely, proton pump inhibitors (PPIs; ATC: A02BC01‐07), statin (ATC: C10), and selective serotonin reuptake inhibitors (selective serotonin reuptake inhibitors [SSRIs]; ATC: N06AB) [31], was additionally considered by including a binary variable indicating the history of prescribing any of them within 1 year before the index date.

### Statistical analysis

First, we compared baseline characteristics between MC patients and their comparators and visualized cumulative hazards of all‐cause dementia and each dementia subtype.

Second, prospective association of MC with incident dementia was examined via Cox models for population comparison and stratified Cox models for sibling comparison, using time since index date as underlying timescale and adjusting for aforementioned covariates [32]. Importantly, as Schoenfield residuals test suggested violation of the proportional hazards assumption over the entire follow‐up period, we estimated time‐varying associations in two ways: (1) We cut the study time into three time‐bands, that is, (0, 5] or 0–5 years, (5, 10] or 5–10 years, and >10 years since index date, and estimated time‐band‐specific associations; (2) flexible parametric models were used to visualize the smoothed association curves [33].

Next, retrospective association with pre‐index dementia was estimated by logistic models using population and sibling comparison, separately, adjusting for the same set of covariates. To see if this association may be explained by pre‐index use of three MC‐related medications, a sensitivity analysis of subsamples indexed 2006–2021 was supplemented to additionally account for prescription history. For comparative analysis purpose, we also performed a post hoc study of the association of IBD, a gastrointestinal disease with similar symptoms as MC [22], and prior dementia using a similar modeling strategy.

Throughout the study, subgroup analyses were implemented by dementia (AD and VaD) and MC subtypes (CC and LC). *p‐*Values <0.05 from a two‐sided test were considered statistically significant. Analyses were performed in R version 4.3.1.

## Results

### Sample characteristics

**Fig. 1:**
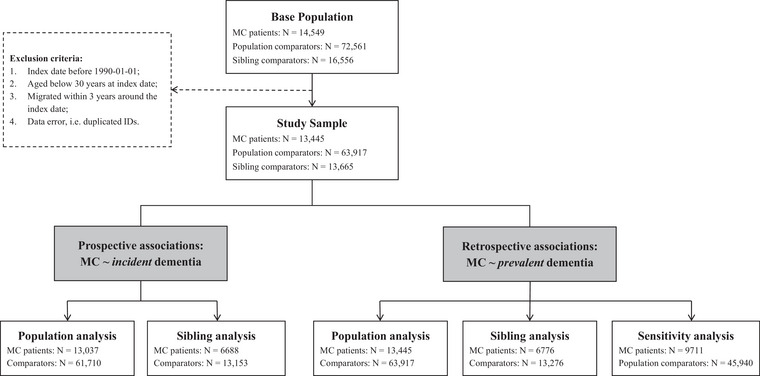
Flowchart of the study. MC, microscopic colitis.

We followed 13,037 MC patients (33.3% CC) and 61,710 population comparators for incident dementia diagnosis (Fig. 1, Table 1). Due to matching, index age and sex were distributed similarly across MC patients and their comparators. As expected, MC patients had a higher prevalence of each selected comorbidity and more healthcare visits than population comparators. No difference in mortality or educational level was observed. Characteristics for CC versus LC patients were also comparable, except that the former had a higher percentage of females and less‐educated patients. Overall, compared to the population sample, the sibling sample (*N*_patients_/*N*_comparators_: 6688/13,153) was 3–4 years younger and better educated (Table 2). Compared to the population comparators, MC‐free siblings were more gender‐balanced and had a greater burden of comorbidities (11.5% vs. 9.5%), particularly IBD (1.4% vs. 0.2%).

**Table 1:** Characteristics of microscopic colitis patients and their matched unaffected population comparators.

	Population comparator	Patients		
		MC	CC	LC
*N*	61,710 (100)	13,037 (100)	4345 (100)	8692 (100)
Age at index date	63.5 [53.0–72.5]	64.1 [53.4–73.4]	65.5 [55.9–74.5]	63.2 [52.2–72.7]
Male	17,119 (27.7)	3617 (27.7)	996 (22.9)	2621 (30.2)
Nordic born	55,489 (89.9)	12,305 (94.4)	4175 (96.1)	8130 (93.5)
Years of follow‐up	10.1 [6.9–14.2]	9.7 [6.6–13.8]	9.7 [6.6–13.6]	9.8 [6.6–13.9]
Person‐years during follow‐up, ×10^5^	6.63	1.34	0.44	0.90
Mortality rate	1.22 (1.20, 1.24)	1.23 (1.19, 1.27)	1.22 (1.16, 1.29)	1.23 (1.19, 1.28)
Hospital visits	1 [0–4]	3 [1–8]	3 [1–8]	3 [1–8]
Incident dementia diagnosed after index date				
All‐cause dementia	3791 (6.1)	883 (6.8)	314 (7.2)	569 (6.5)
Alzheimer's disease	1749 (2.8)	408 (3.1)	138 (3.2)	270 (3.1)
Vascular dementia	797 (1.3)	199 (1.5)	67 (1.5)	132 (1.5)
Other dementia	2328 (3.8)	513 (3.9)	190 (4.4)	323 (3.7)
Years of education at index date				
≤9	16,222 (26.3)	3484 (26.7)	1314 (30.2)	2170 (25.0)
10–12	25,588 (41.5)	5407 (41.5)	1812 (41.7)	3595 (41.4)
≥13	18,710 (30.3)	3907 (30.0)	1130 (26.0)	2777 (31.9)
Missing	1190 (1.9)	239 (1.8)	89 (2.0)	150 (1.7)
Prevalent comorbidities				
COPD	1386 (2.2)	505 (3.9)	206 (4.7)	299 (3.4)
Unipolar depression	2658 (4.3)	1182 (9.1)	395 (9.1)	787 (9.1)
Anxiety	1743 (2.8)	763 (5.9)	260 (6.0)	503 (5.8)
IBD	110 (0.2)	314 (2.4)	120 (2.8)	194 (2.2)
Type 1 diabetes	1100 (1.8)	405 (3.1)	174 (4.0)	231 (2.7)
Any of above	5882 (9.5)	2517 (19.3)	916 (21.1)	1601 (18.4)

*Note*: Values are *N* (%) for categorical variables, point estimate (95% confidence interval) for mortality rate (per 100 person‐years), and median (interquartile range) for age at index date, years of follow‐up, hospital visits recorded within 3 years to 6 months before the index date.

Abbreviations: CC, collagenous colitis; COPD, chronic obstructive pulmonary disease; IBD, inflammatory bowel disease; LC, lymphocytic colitis; MC, microscopic colitis.

**Table 2:** Characteristics of microscopic colitis patients and their unaffected siblings.

	MC patients	Sibling comparators
*N*	6688 (100)	13153 (100)
Age at index date	59.3 [49.8–66.7]	58.9 [50.2–66.1]
Male	1855 (27.7)	6544 (49.8)
Born in Nordic countries	6629 (99.1)	13,054 (99.2)
Years of follow‐up	10.5 [7.4–14.5]	10.7 [7.7–14.7]
Person‐years during follow‐up, ×10^5^	0.75	1.51
Mortality rate, per 100PYs	1.36 (1.28, 1.44)	1.36 (1.30, 1.42)
Hospital visits	3 [1–8]	1 [0–4]
Incident dementia diagnosed after index date		
All‐cause dementia	237 (3.5)	353 (2.7)
Alzheimer's disease	134 (2.0)	206 (1.6)
Vascular dementia	41 (0.6)	81 (0.6)
Other dementia	110 (1.6)	163 (1.2)
Years of education at index date		
≤9	1312 (19.6)	2978 (22.6)
10–12	3011 (45.0)	5943 (45.2)
≥13	2331 (34.9)	4156 (31.6)
Missing	34 (0.5)	76 (0.6)
Prevalent comorbidities		
COPD	192 (2.9)	297 (2.3)
Unipolar depression	614 (9.2)	633 (4.8)
Anxiety	448 (6.7)	467 (3.6)
Inflammatory bowel disease	178 (2.7)	178 (1.4)
Type 1 diabetes	219 (3.3)	267 (2.0)
Any of above	1293 (19.3)	1519 (11.5)

*Note*: Values are *N* (%) for categorical variables, point estimate (95% confidence interval) for mortality rate (per 100 person‐years), and median (interquartile range) for age at index date, years of follow‐up, hospital visits recorded within 3 years to 6 months before the index date.

Abbreviations: COPD, chronic obstructive pulmonary disease; MC, microscopic colitis.

The population and sibling samples analyzed in the retrospective analysis of prevalent dementia are summarized in Table S2. Notably, all‐cause dementia was three‐time more prevalent in the population (3.5%) than in the sibling sample (0.9%). Compared to MC cases, pre‐index dementia was less frequently diagnosed in sibling controls (0.9% vs. 1.3%) but more common among population controls (3.5% vs. 3.0%). Among the subsample indexed 2006–2017 (Table S3), PPIs, statin, and SSRIs were all more commonly consumed by MC cases than their population controls regardless of MC subtype.

### MC and subsequent diagnosis of dementia

In the population sample, 883 (6.8%) MC patients and 3791 (6.1) comparators developed incident dementia, 46% of which were AD (Table 1). In the sibling sample, incident dementia was diagnosed for 237 (3.5%) MC patients and 353 (2.7%) unaffected siblings (Table 2). These yielded a crude incidence rate ratio for dementia of 1.02 for population comparison and 0.99 for sibling comparison. Over the follow‐up time, cumulative hazards of all‐cause dementia were higher among MC patients than population comparators (Fig. S1a) and the contrast seemed more prominent in the sibling sample (Fig. S1b).

**Fig. 2:**
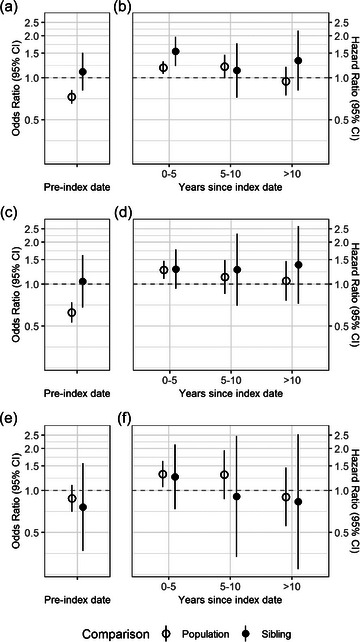
Bidirectional associations between microscopic colitis and dementia by subtype. Associations with prevalent (a) and incident (b) diagnosis of all‐cause dementia, with prevalent (c) and incident diagnosis of Alzheimer's disease (d), and with prevalent (e) and incident diagnosis of vascular dementia (f). Odds ratios (a, c, and e) were estimated from medication‐unadjusted logistic models (for population comparison) or conditional logistic models (for sibling comparison) including index age, sex, index year, educational attainment, birth country (only for population comparison), comorbidity, and pre‐index hospital visit as covariates. Hazard ratios (b, d, and f) were estimated from Cox models (for population comparison) or stratified Cox models (for sibling comparison) splitting years since index date and adjusting for the same set of covariates listed above. CI, confidence interval.

After multivariate adjustment, MC was associated with a 19% higher risk of all‐cause dementia (hazard ratio [HR]: 1.19; 95% confidence interval [CI]: 1.07–1.32) within the first 5 years of study (Fig. 2b, Table 3). The association remained positive at the same magnitude for another 5 years (HR: 1.20; 95% CI: 0.99–1.46) and then declined to null (HR: 0.95; 95% CI: 0.75–1.20). Such temporal patterns were observed for both dementia subtypes, although HRs in the first 10 years tended to be stronger for VaD (Fig. 2f) versus AD (Fig. 2d). In general, the main findings based on population comparison were replicated in our sibling analysis, except that the short‐term positive association attenuated more rapidly immediately after the first 5 years and did not continue to drop beyond 10 years since index date. These temporal patterns of the MC association with incident dementia were also consistent with the smoothed curves for adjusted HRs estimated via flexible parametric models (Fig. S2).

**Table 3:** Bidirectional associations of microscopic colitis with dementia and its subtypes.

	Odds ratio^a^ (95% CI)	Hazard ratio (95% CI)^b^		
		0–5 years	5–10 years	>10 years
Population comparison				
All‐cause dementia	0.73 (0.65, 0.82)	1.19 (1.07, 1.32)	1.20 (0.99, 1.46)	0.95 (0.75, 1.20)
Alzheimer's disease	0.63 (0.53, 0.74)	1.26 (1.09, 1.47)	1.13 (0.85, 1.49)	1.06 (0.76, 1.46)
Vascular dementia	0.88 (0.71, 1.10)	1.31 (1.06, 1.63)	1.30 (0.87, 1.94)	0.90 (0.56, 1.46)
Sibling comparison				
All‐cause dementia	1.11 (0.81, 1.51)	1.55 (1.22, 1.97)	1.13 (0.72, 1.77)	1.33 (0.81, 2.19)
Alzheimer's disease	1.05 (0.68, 1.62)	1.28 (0.93, 1.78)	1.27 (0.70, 2.30)	1.38 (0.73, 2.61)
Vascular dementia	0.76 (0.37, 1.57)	1.25 (0.74, 2.14)	0.91 (0.33, 2.47)	0.83 (0.27, 2.54)

^a^ Association of microscopic colitis with prior diagnosis of dementia estimated from medication‐unadjusted logistic models (for population comparison) or conditional logistic models (for sibling comparison), including index age, sex, index year, educational attainment, birth country (only for population comparison), comorbidity, and pre‐index hospital visit as covariates.

^b^ Association of microscopic colitis with subsequent diagnosis of dementia estimated from Cox models (for population comparison) or stratified Cox models (for sibling comparison) splitting years since index date and adjusting for the same set of covariates listed above.

### MC and prevalent diagnosis of dementia

Compared to population controls, MC cases had a 27% lower prevalence of all‐cause dementia (odds ratio [OR]: 0.73; 95% CI: 0.65–0.82), which seemed to be mainly attributed to a lack of AD relative to VaD (Fig. 2a, Table 3). This inverse association remained nearly unchanged when history of prescribing MC‐related medications was further accounted for (Table S3); however, it was not supported by sibling results (OR: 1.11; 95% CI: 0.81–1.51) regardless of dementia subtype. In a post hoc comparative analysis, IBD patients were also less frequently diagnosed with dementia of any subtype than the general population (Table S4).

### Association by MC subtype

Subgroup analyses by MC subtype delineated that the short‐term association with 5‐year risk of all‐cause dementia was driven more by the extra diagnoses following CC (HR: 1.32; 95% CI: 1.12–1.55) than LC (HR: 1.12; 95% CI: 0.99–1.27) (Table S5, Fig. S3). Particularly for VaD, the risk was 60% higher (HR: 1.60; 95% CI: 1.16–2.20) in CC patients versus general population and was more than doubled (HR: 2.21; 95% CI: 1.04–4.70) when full siblings were compared to. The 5‐year risk of AD was also significantly associated with CC in both population and sibling comparisons. In contrast, LC patients had non‐differential risk of VaD compared to population and sibling comparators throughout the disease course, and the short‐term HR for AD appeared weaker and was only marginally significant in population analysis. Interestingly, the inverse association with prevalent diagnosis of all‐cause dementia also appeared more prominent for CC (OR: 0.64; 95% CI: 0.54–0.77) than LC (OR: 0.79; 95% CI: 0.69–0.91), despite the overlap in CIs. Nevertheless, as seen in the overall analysis of MC, neither of these signals were replicated by the sibling analysis.

## Discussion

This nationwide study of more than 13,000 histologically confirmed MC patients and their matched comparators in Sweden provides the first epidemiologic evidence for a bidirectional relation between MC and dementia. Overall, we demonstrate a robust elevation of dementia risk during the first 5 years following MC diagnosis. We also detect a significantly lower burden of dementia history, particularly AD, among MC patients versus general population.

Although research on dementia risk and cognition in MC population remains scarce, multiple teams have documented an increased risk of dementia following IBD, an intestinal inflammatory disease closely linked to MC [22]. A recent meta‐analysis of ∼110,000 IBD patients from five cohort studies delineated a pooled risk ratio of all‐cause dementia at 1.30 (95% CI: 1.09–1.55) [23], supporting the emerging findings about multidomain neurocognitive deficits [34, 35] and brain structural alterations [36, 37] in IBD. In consistency, we showed that MC associates with a higher risk of dementia during the early disease course. This signal remained statistically significant and became stronger in our sibling analysis that implicitly addressed unmeasured familial confounding, for example, genetics and shared early‐life environmental factors. Toward later disease course, dementia risk was no longer differential between MC patients and their population or sibling comparators. This may be explained by the possibility that dementia‐susceptible subjects were more likely to be diagnosed with dementia and removed from subsequent follow‐up from MC versus non‐MC group, leading to an increasingly lower enrichment of dementia susceptibility in MC versus non‐MC group and the resulted attenuation in the estimated association over study period [38]. Future work is thus warranted to scrutinize the MC association with long‐term dementia appropriately accounting for the time‐specific dementia susceptibility in the study sample.

Another important discovery is the inverse association of MC with prevalent dementia independent from the consumption of medications commonly linked to MC onset. Intuitively, it suggests a possible underdiagnosis of MC among demented patients, which may be due to the cognitive deficits or deprivation affecting this vulnerable population and the resulted failure to present their symptoms to caregivers. To test this hypothesis, we carried out a post hoc analysis comparing dementia history between subjects with and without IBD, considering its symptomatic similarity to MC. As speculated, a significantly smaller proportion of IBD patients suffered from dementia than the population controls. Yet when sibling comparison was implemented, neither MC nor IBD associated with previous diagnosis of AD or VaD. Hence, whether demented patients are indeed under healthcare disparities in diagnosis of MC and other gastrointestinal conditions remains to be clarified. As MC can severely impact quality of life [39], addressing MC symptoms could substantially benefit this already vulnerable population.

Biologically, our novel finding about the MC association with dementia may be underlined by several plausible mechanisms. First, MC is characterized by mucosal inflammation, and many pro‐inflammatory cytokines, such as tumor necrosis factor *α* and interleukin‐6, have been shown at elevated level in the colonic tissues of MC patients [1]. Notably, these cytokines are also implicated in neuroinflammation and, via the blood–brain‐barrier dysfunction, neurodegeneration that typically attribute to dementia [40, 41]. Second, intestinal dysbiosis observed in MC may alter microbial metabolites and similar changes have also been implicated in AD pathology [9, 42]. Besides, the commonly reported coexistence of MC with autoimmune [43, 44, 45] and vascular diseases [46] further implies a potential contribution of autoimmune and endothelial dysfunctions to the pathogenesis of both MC and dementia [47]. Hence, despite the lack of direct mechanistic evidence, multiple pathogenic pathways have been found to overlap between MC and dementia.

Key strength of the work includes the nationwide study design that maximized statistical power and result generalizability. Additionally, Sweden's tax‐funded healthcare system, designed to provide equal access to care regardless of socioeconomic status, minimizes selection bias related to financial factors. The use of the Swedish personal identity number [48] also ensured complete follow‐up of all participants across multiple registers, further strengthening the study's robustness. The investigation of both prevalent and incident dementia in MC as well as the consideration of a time‐varying association of MC with short‐ and long‐term dementia risk further enhanced the comprehensiveness and resolution of study findings. In addition, MC status was ascertained from the nationwide pathology database in Sweden, which has been validated and has a 95% positive predictive value for clinical MC [2]. An evidence‐based identification of register diagnosis of dementia, particularly the adjustment of the date of diagnosis, was also implemented to minimize ascertainment bias [18, 19, 20]. Lastly, throughout the analysis, we included a carefully selected list of covariates and justified the robustness of the main findings via various sensitivity analyses.

There are also limitations. First, despite our effort to ascertain dementia from multiple registers, misclassification of dementia due to missing dementia cases diagnosed in primary care may still distort our estimation. Yet this misclassification is likely to be non‐differential as it falls on primary care to initiate evaluation of dementia in Sweden. If MC patients may be more frequently referred by their gastroenterologists to the primary care for workup due to more regular healthcare contact, which would shift the misclassification toward an issue of surveillance bias, we also adjusted for the pre‐index hospital visits throughout the study. Misclassification between dementia subtypes is also possible; however, investigation on dementia by subtype is of secondary interest only. Moreover, although all efforts have been made to account for the known delay in register‐based diagnosis, we also acknowledge the insufficient accuracy in date of dementia diagnosis and have thus presented time‐specific HRs throughout to facilitate interpretations. Second, our estimates for the MC association with long‐term dementia risk may be subject to selection bias inherent to the approach of time‐specific HR estimation [38]. To visualize the impact of this potential bias, we plotted the time‐varying associations also using flexible parametric models. Meanwhile, given the continuous rise of dementia incidence among the oldest‐old (i.e., 85+ years), our capacity to capture an even longer‐term dementia risk following MC may be restricted by the current study duration, which allowed us to follow nearly one third of all participants until 80 years or older at study end. However, our time‐specific findings have revealed a consistent and monotonic decline in HRs over time and implied that MC patients may be at higher risk of earlier dementia onset. Assuming this trend continues and in the absence of evidence suggesting a reversal (i.e., a worsening or recurring MC pathology contributing to dementia initiation after 15 or more years since its initial diagnosis), it is reasonable to hypothesize that dementia risk beyond 14 years, the maximum of our current study follow‐up, may remain non‐differential between MC patients and their population comparators. Third, our results may subject to residual confounding. For instance, due to the lack of smoking record in the register data, we only included COPD as a proxy for heavy smoking for adjustment. Similarly, we were also not able to account for obesity or body mass index (BMI) due to unavailability. Nevertheless, this could only have biased our estimates toward null, as BMI or weight gain is inversely associated with MC risk, but obesity or a higher BMI, particularly in mid‐life, is a risk factor for dementia. Last, our sibling analysis may lack power to test for replication of our population results, especially in subgroup analyses.

In conclusion, this nationwide study demonstrates a bidirectional association between MC and dementia. The increased dementia risk observed in early phase of MC underscores the need for heightened clinical awareness for mild cognitive deficit among physicians treating MC patients, as early detection and management of dementia can significantly benefit patients. The lower prevalence of earlier dementia in MC also implies a potential underdiagnosis of MC in dementia patients, which highlights the importance of maintaining vigilance in this vulnerable population. Further investigation is warranted to replicate our findings and to unravel the pathological implications underlying this relationship.

## Author contributions

Xiaoying Kang, David Bergman, and Jonas F. Ludvigsson contributed to the conception, organization, and execution of the study. Xiaoying Kang and David Bergman contributed to the design and execution of statistical analysis. The manuscript was drafted by Xiaoying Kang and David Bergman All authors reviewed and critiqued the results of statistical analysis and the manuscript.

## Conflict of interest statement

J.F.L. has coordinated a study on behalf of the Swedish IBD quality register (SWIBREG). That study received funding from Janssen Corporation. J.F.L. has also received financial support from Merck for an unrelated study on inflammatory bowel disease, and for developing a paper reviewing national healthcare registers in China. He has also an ongoing study on celiac disease funded by Takeda.

## Funding information

This work was supported by the Karolinska Institutet (J.F.L.) and Stockholm County Council (J.F.L.). X.K. was funded by the Swedish Research Council International Postdoc Grant (2022‐00164), the Swedish Parkinson Foundation, Loo och Hans Ostermans Stiftelse, and KI Research Foundation. J.S. was supported by the European Crohn's and Colitis Organization and the Swedish Society for Medical Research. K.W. was supported by Region Stockholm (clinical research appointment). None of the funding organizations has had any role in the design and conduct of the study, in the collection, management, and analysis of the data, or in the preparation, review, and approval of the manuscript.

## Ethics statement

The study was approved by the Regional Ethics Review Board, Stockholm, Sweden (Diarienummer: 2014/1287‐31/4).

## Supporting information




**Table S1**: The Swedish‐version International Classification of Diseases (ICD) and Anatomical Therapeutic Chemical (ATC) codes used in the present study.
**Table S2**: Baseline characteristics of samples included in the matched case‐control analyses.
**Table S3**: Medication‐adjusted association of microscopic colitis with prevalent dementia among the sub‐sample with index date between 2006 and 2017.
**Table S4**: Associations of inflammatory bowel disease with prevalent dementia.
**Table S5**: Bidirectional associations of collagenous colitis and lymphocytic colitis, respectively, with dementia and its subtypes.
**Fig. S1**: Cumulative incidence of dementia and its subtypes among patients with microscopic colitis and their comparators.
**Fig. S2**: Adjusted hazard ratios for MC association with dementia and its subtypes estimated from flexible parametric models.
**Fig. S3**: Bidirectional associations between collagenous colitis and lymphocytic colitis, respectively, and dementia by subtype.

## Data Availability

The ESPRESSO data analyzed in this study are available through Swedish pathology departments. The original register data analyzed in this study are held by the Swedish National Board of Health and Welfare and Statistics Sweden and we cannot make the data available due to Swedish data privacy laws. Any researcher can get access to the register data by obtaining an ethical approval from a regional ethical review board and then make a request to the Swedish National Board of Health and Welfare and Statistics Sweden. X.K. takes responsibility for the integrity of the data and the accuracy of the data analysis.
