# Supplementary material for: Microscopic colitis is associated with an increased risk of dementia in a Swedish population
Source: J Intern Med. 2025 Nov 26;299(2):216–27. doi: 10.1111/joim.70046 (PMC12789275; doi:10.1111/joim.70046)

**Title:**

Association between Microscopic Colitis and Dementia in a Swedish Population

**Supplementary Materials**

**Table S1**. The Swedish-version International Classification of Diseases (ICD) and Anatomical Therapeutic Chemical (ATC) codes used in the present study.

**Table S2**. Baseline characteristics of samples included in the matched case-control analyses.

**Table S3**. Medication-adjusted association of microscopic colitis with prevalent dementia among the sub-sample with index date between 2006-2017.

**Table S4**. Associations of inflammatory bowel disease with prevalent dementia.

**Table S5**. Bidirectional associations of collagenous colitis and lymphocytic colitis, respectively, with dementia and its subtypes.

**Figure S1.** Cumulative incidence of dementia and its subtypes among patients with microscopic colitis and their comparators.

**Figure S2**. Adjusted hazard ratios for MC association with dementia and its subtypes estimated from flexible parametric models.

**Figure S3**. Bidirectional associations between collagenous colitis and lymphocytic colitis, respectively, and dementia by subtype.

**Table S1**. The Swedish-version International Classification of Diseases (ICD) and Anatomical Therapeutic Chemical (ATC) codes used in the present study.

|  | Source data | Codes |
| --- | --- | --- |
| - *Dementia* | | |
| Alzheimer’s disease | NPR or CDR | **ICD-07**: 304 – 305  **ICD-08**: 290  **ICD-09**: 290A/B/X, 331A  **ICD-10**: F00, G30 |
|  | PDR | **ATC**: N06DA02 – N06DA04, N06DX01 |
| Vascular dementia | NPR or CDR | **ICD-07**: 306  **ICD-08**: 293.0 – 293.1  **ICD-09**: 290E  **ICD-10**: F01 |
| Other dementia | NPR or CDR | **ICD-09**: 294B, 290W, 331B/C/X  **ICD-10**: F02, F03, F05.1, G31.1, G31.8 |
| - *Comorbidities* | | |
| Inflammatory bowel disease | NPR | **ICD-07**: 572.00, 572.09, 572.20, 572.21, 572.30  **ICD-08**: 563.00, 563.10, 569.02, 563.99  **ICD-09**: 555, 556  **ICD-10**: K50, K51  *Requiring at least two ICD-based diagnoses. |
| Type 1 diabetes | NPR | **ICD-07**: 260  **ICD-08**: 250  **ICD-09**: 250  **ICD-10**: E10  *Requiring age at diagnosis ≤30 years for diagnoses identified via ICD-07, ICD-08 or ICD-09. |
| Chronic obstructive pulmonary disease | NPR | **ICD-08**: 491, 492  **ICD-09**: 491, 492, 496  **ICD-10**: J41 – J44 |
| Unipolar depression | NPR | **ICD-08**: 300.4  **ICD-09**: 296B, 300E, 311  **ICD-10**: F32, F33, F34, F39 |
|  | PDR | **ATC**: N06AB |
| Anxiety | NPR | **ICD-8:** 300.00, 300.20, 300.30, 307  **ICD-9:** 300A, 300C, 300D, 300X  **ICD-10:** F40, F41, F42 |
| *Abbreviations*: CDR, Cause of Death Register; NPR, National Patient Register; PDR, Prescribed Drug Register. | | |

**Table S2**. Pre-index characteristics of microscopic colitis cases and their population and sibling comparators analyzed in the retrospective analysis.

|  | Population analysis | | Sibling analysis | |
| --- | --- | --- | --- | --- |
|  | Cases | Controls | Cases | Controls |
| N | 13,445 (100) | 63,917 (100) | 6776 (100) | 13,276 (100) |
| Age at index date | 64.6  [53.8 – 74.0] | 64.1  [53.5 – 73.4] | 59.5  [50.0 – 66.9] | 59.0  [50.3 – 66.3] |
| Male | 3747 (27.9) | 17,719 (27.7) | 1887 (27.8) | 6596 (49.7) |
| Nordic born | 12,700 (94.5) | 57,577 (90.1) | 6716 (99.1) | 13,177 (99.3) |
| Lead-time in years^*^ | 2.1 [1.0 – 3.5] | 3.3 [1.7 – 5.3] | 2.3 [1.0 – 4.6] | 2.8 [1.1 – 5.1] |
| Hospital visits | 3 [1 - 8] | 1 [0 - 4] | 3 [1 - 8] | 1 [0 - 4] |
| *Pre-index diagnosis of dementia* | | | | |
| All-cause dementia | 408 (3.0) | 2207 (3.5) | 88 (1.3) | 123 (0.9) |
| Alzheimer’s disease | 196 (1.5) | 1149 (1.8) | 43 (0.6) | 71 (0.5) |
| Vascular dementia | 112 (0.8) | 509 (0.8) | 18 (0.3) | 28 (0.2) |
| Other dementia | 257 (1.9) | 1476 (2.3) | 56 (0.8) | 68 (0.5) |
| *Years of education at index date* | | | | |
| ≤ 9 | 3669 (27.3) | 17,191 (26.9) | 1333 (19.7) | 3009 (22.7) |
| 10 – 12 | 5536 (41.2) | 26,278 (41.4) | 3054 (45.1) | 6002 (45.2) |
| ≥ 13 | 3983 (29.6) | 19,122 (29.9) | 2354 (34.7) | 4189 (31.6) |
| Missing | 257 (1.9) | 1326 (2.1) | 35 (0.5) | 76 (0.6) |
| *Prevalent comorbidities* | | | | |
| COPD | 541 (4.0) | 1494 (2.3) | 200 (3.0) | 304 (2.3) |
| Unipolar depression | 1253 (9.3) | 2905 (4.5) | 636 (9.4) | 653 (4.9) |
| Anxiety | 792 (5.9) | 1835 (2.9) | 460 (6.8) | 477 (3.6) |
| IBD | 320 (2.4) | 115 (0.2) | 178 (2.6) | 179 (1.3) |
| Type 1 diabetes | 429 (3.2) | 1198 (1.9) | 225 (3.3) | 271 (2.0) |
| Any of above | 2640 (19.6) | 6342 (9.9) | 1324 (19.5) | 1550 (11.7) |
| ^*^Calculated as years between the diagnosis of pre-existing dementia and the index date for those with a history of dementia.  Values are N (%) for categorical variables, and median (interquartile range) for age at index date, lead-time in years and hospital visits recorded within 3 years to 6 months before the index date.  *Abbreviations*: COPD, chronic obstructive pulmonary disease; IBD, inflammatory bowel disease. | | | | |

**Table S3**. Medication-adjusted association of microscopic colitis with prevalent dementia among the sub-sample with index date between 2006-2017.

|  | Cases | | | Population controls |
| --- | --- | --- | --- | --- |
|  | Microscopic colitis | Collagenous colitis | Lymphocytic colitis |  |
| N (%) | 9711 (100) | 3229 (100) | 6482 (100) | 45,940 (100) |
| *Number of pre-index conditions, N (%)* | | | | |
| All-cause dementia | 303 (3.1) | 111 (3.4) | 192 (3.0) | 1568 (3.4) |
| PPI | 3049 (31.4) | 1156 (35.8) | 1893 (29.2) | 5177 (11.3) |
| Statin | 2569 (26.5) | 963 (29.8) | 1606 (24.8) | 7530 (16.4) |
| SSRIs | 2094 (21.6) | 612 (19.0) | 1482 (22.9) | 3673 (8.0) |
| Any of PPIs, statin, or SSIs | 5560 (57.3) | 1941 (60.1) | 3619 (55.8) | 13,560 (29.5) |
| *Odds ratio (95% CI) from medication-adjusted models^1^* | | | | |
| All-cause dementia | 0.72 (0.63, 0.83) | 0.66 (0.54, 0.81) | 0.77 (0.66, 0.90) | Reference |
| Alzheimer’s disease | 0.60 (0.50, 0.73) | 0.47 (0.34, 0.65) | 0.70 (0.55, 0.87) | Reference |
| Vascular disease | 0.82 (0.62, 1.07) | 0.71 (0.46, 1.08) | 0.92 (0.66, 1.27) | Reference |
| ^1^Estimated from medication-adjusted logistic models including index age, sex, index year, educational attainment, birth country, comorbidity, pre-index hospital visit as well as pre-index history of prescribing any of PPIs, SSRIs or statin as covariates.  *Abbreviations*: PPI, proton pump inhibitors; SSRI, selective serotonin reuptake inhibitors. | | | | |

**Table S4**. Associations of inflammatory bowel disease with prevalent dementia and its subtypes.

|  | Population analysis | Sibling analysis |
| --- | --- | --- |
| N_cases_/N_controls_ | 56,788/281,488 | 32,712/64,015 |
| Number of prevalent diagnoses | 4918 | 471 |
| *Odds ratio (95% CI) by dementia subtype^1^* | | |
| All-cause dementia | 0.70 (0.64, 0.76) | 0.74 (0.57, 0.94) |
| Alzheimer’s disease | 0.69 (0.58, 0.83) | 0.62 (0.39, 0.99) |
| Vascular dementia | 0.63 (0.49, 0.81) | 0.68 (0.35, 1.34) |
| ^1^Estimated from the medication-unadjusted logistic models (for population analysis) or conditional logistic models (for sibling analysis)including index age, sex, index year, educational attainment, birth country (only for population analysis), comorbidity and pre-index hospital visit as covariates. | | |

**Table S5**. Bidirectional associations of collagenous colitis and lymphocytic colitis with dementia and its subtypes.

|  | | Collagenous colitis | | Lymphocytic colitis | |
| --- | --- | --- | --- | --- | --- |
|  |  | Population | Sibling | Population | Sibling |
| *Association with all-cause dementia* | | | | | |
| OR (95% CI)^1^ | | **0.64**  **(0.54, 0.77)** | 1.04  (0.59, 1.81) | **0.79**  **(0.69, 0.91)** | 1.14  (0.78, 1.66) |
| HR  (95% CI)^2^ | 0 - 5 | **1.32**  **(1.12, 1.55)** | **1.83**  **(1.20, 2.77)** | 1.12  (0.99, 1.27) | **1.42**  **(1.06, 1.92)** |
|  | 5-10 | 1.22  (0.90, 1.66) | 1.07  (0.50, 2.33) | 1.19  (0.95, 1.50) | 1.16  (0.67, 2.01) |
|  | >10 | 1.07  (0.74, 1.54) | 1.24  (0.54, 2.86) | 0.89  (0.67, 1.18) | 1.40  (0.75, 2.59) |
| *Association with Alzheimer’s disease* | | | | | |
| OR (95% CI)^1^ | | **0.47 (0.35, 0.63)** | 0.97 (0.43, 2.21) | **0.73 (0.60, 0.89)** | 1.09 (0.65, 1.81) |
| HR  (95% CI)^2^ | 0 - 5 | **1.40 (1.11, 1.76)** | **1.75 (1.00, 3.05)** | **1.20 (1.00, 1.44)** | 1.10 (0.73, 1.64) |
|  | 5-10 | 1.02 (0.65, 1.61) | 0.84 (0.29, 2.46) | 1.18 (0.85, 1.64) | 1.50 (0.73, 3.08) |
|  | >10 | 1.20 (0.72, 2.00) | 1.45 (0.50, 4.25) | 0.99 (0.66, 1.47) | 1.34 (0.60, 2.99) |
| *Association with vascular dementia* | | | | | |
| OR (95% CI)^1^ | | 0.75  (0.53, 1.07) | 0.63  (0.16, 2.54) | 0.97  (0.74, 1.25) | 0.82  (0.35, 1.90) |
| HR  (95% CI)^2^ | 0 - 5 | **1.60**  **(1.16, 2.20)** | **2.21**  **(1.04, 4.70)** | 1.17  (0.90, 1.53) | 0.72  (0.32, 1.61) |
|  | 5-10 | 1.12  (0.59, 2.13) | 1.05  (0.21, 5.24) | 1.39  (0.86, 2.25) | 0.85  (0.21, 3.41) |
|  | >10 | 0.87  (0.40, 1.90) | 1.10  (0.22, 5.47) | 0.91  (0.51, 1.64) | 0.68  (0.14, 3.38) |
| Results of statistical significance, i.e. with unadjusted *p-value* < 0.05, are highlighted in **bold**.  ^1^Estimated from medication-unadjusted logistic models (for population comparison) or conditional logistic models (for sibling comparison) including index age, sex, index year, educational attainment, birth country (only for population comparison), comorbidity and pre-index hospital visit as covariates.  ^2^Estimated from Cox models (for population comparison) or stratified Cox models (for sibling comparison) splitting years since index date and adjusting for the same set of covariates listed above.  *Abbreviations*: HR, hazards ratio; OR, odds ratio. | | | | | |

**Figure S1.** Cumulative incidence of dementia and its subtypes among patients with microscopic colitis (black line) and their comparators (grey line). A, cumulative incidences of all-cause dementia in MC patients and their population comparators. B, cumulative incidences of all-cause dementia in MC patients and their sibling comparators. C. cumulative incidences of Alzheimer’s disease in MC patients and their population comparators. D. cumulative incidences of vascular dementia in MC patients and their population comparators.


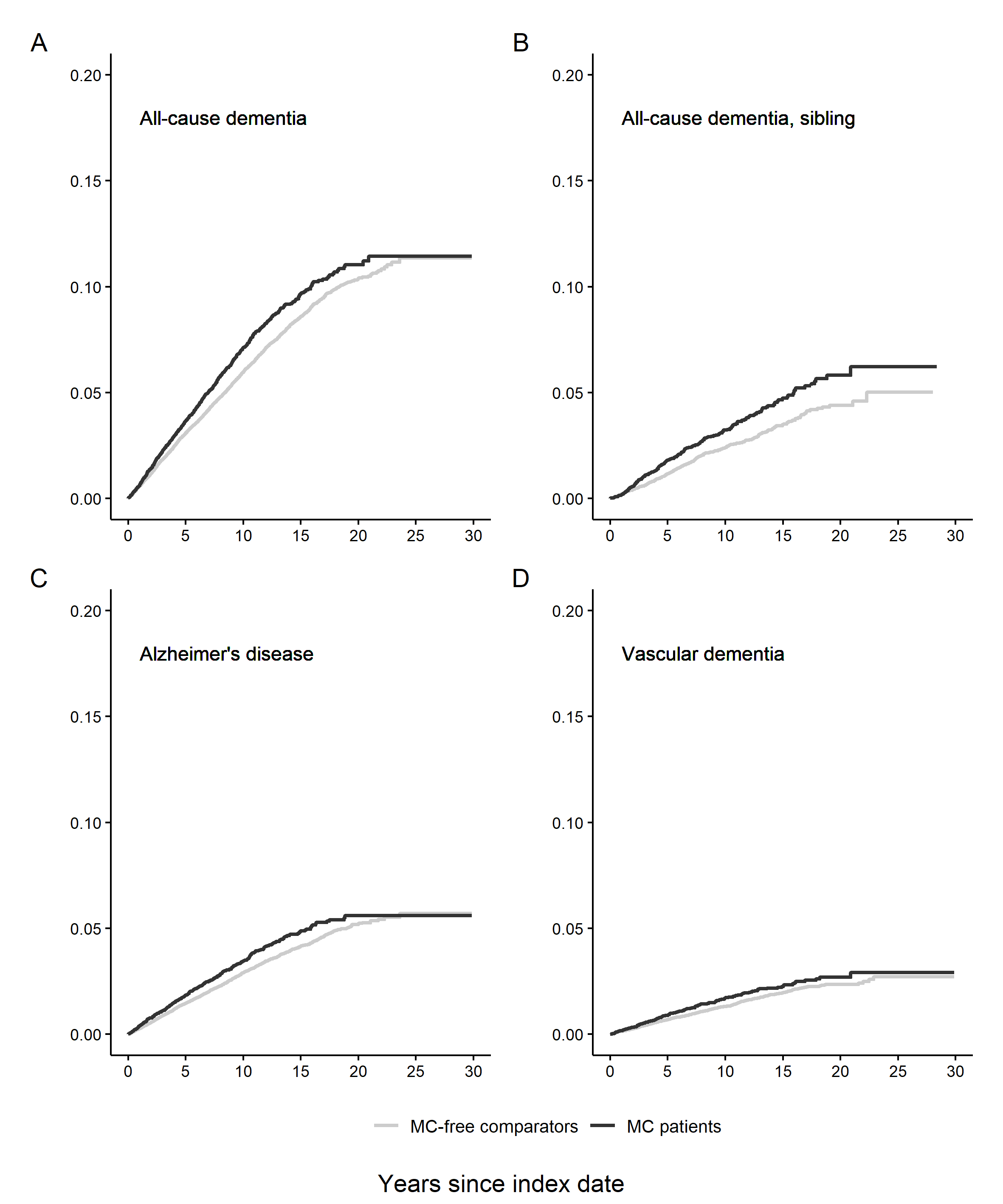


**Figure S2**. Adjusted hazard ratios for MC association with dementia and its subtypes estimated from flexible parametric models including index age (≤50, 50-65, 65-75, or >75), sex, index year (≤2005, 2005-2010, 2010-2013, or >2013), educational attainment, birth country (only for population comparison), comorbidity and pre-index hospital visit (≤1 or >1). A. for all-cause dementia in population comparison. B. for all-cause dementia in sibling comparison. C. for Alzheimer’s disease in population comparison. D. for vascular dementia in population comparison.


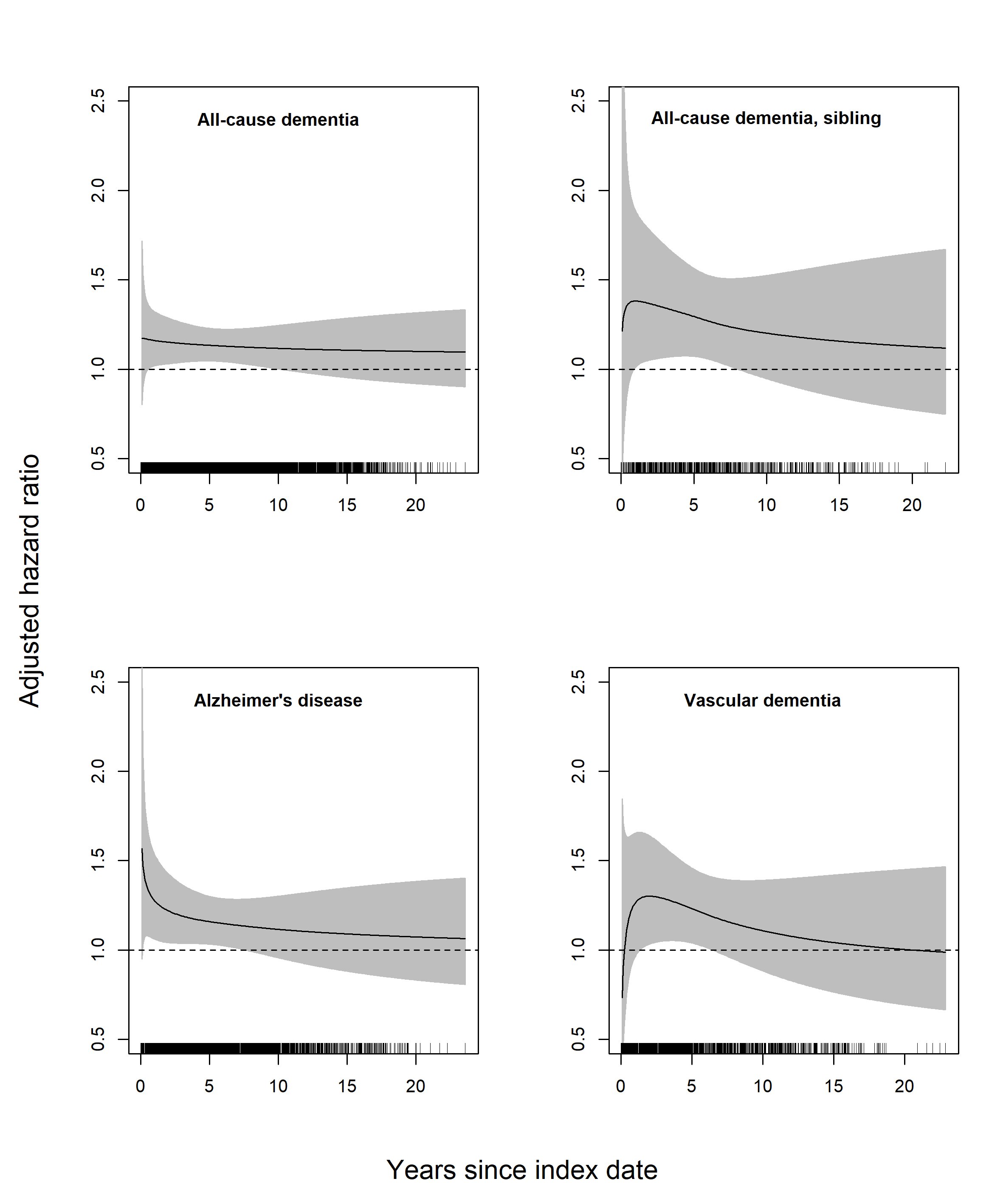


**Figure S3**. Bidirectional associations between collagenous colitis and lymphocytic colitis, respectively, and dementia by subtype. Associations with prevalent (A) and incident diagnosis of all-cause dementia (B), with prevalent (C) and incident diagnosis of Alzheimer’s disease (D), and with prevalent (E) and incident diagnosis of vascular dementia (F). Results were estimated from medication-unadjusted logistic models (for odds ratios) or Cox models (for hazard ratios) including index age, sex, index year, educational attainment, birth country, comorbidity and pre-index hospital visit as covariates. *Abbreviations*: CC, collagenous colitis; CI, confidence interval; LC, lymphocytic colitis.


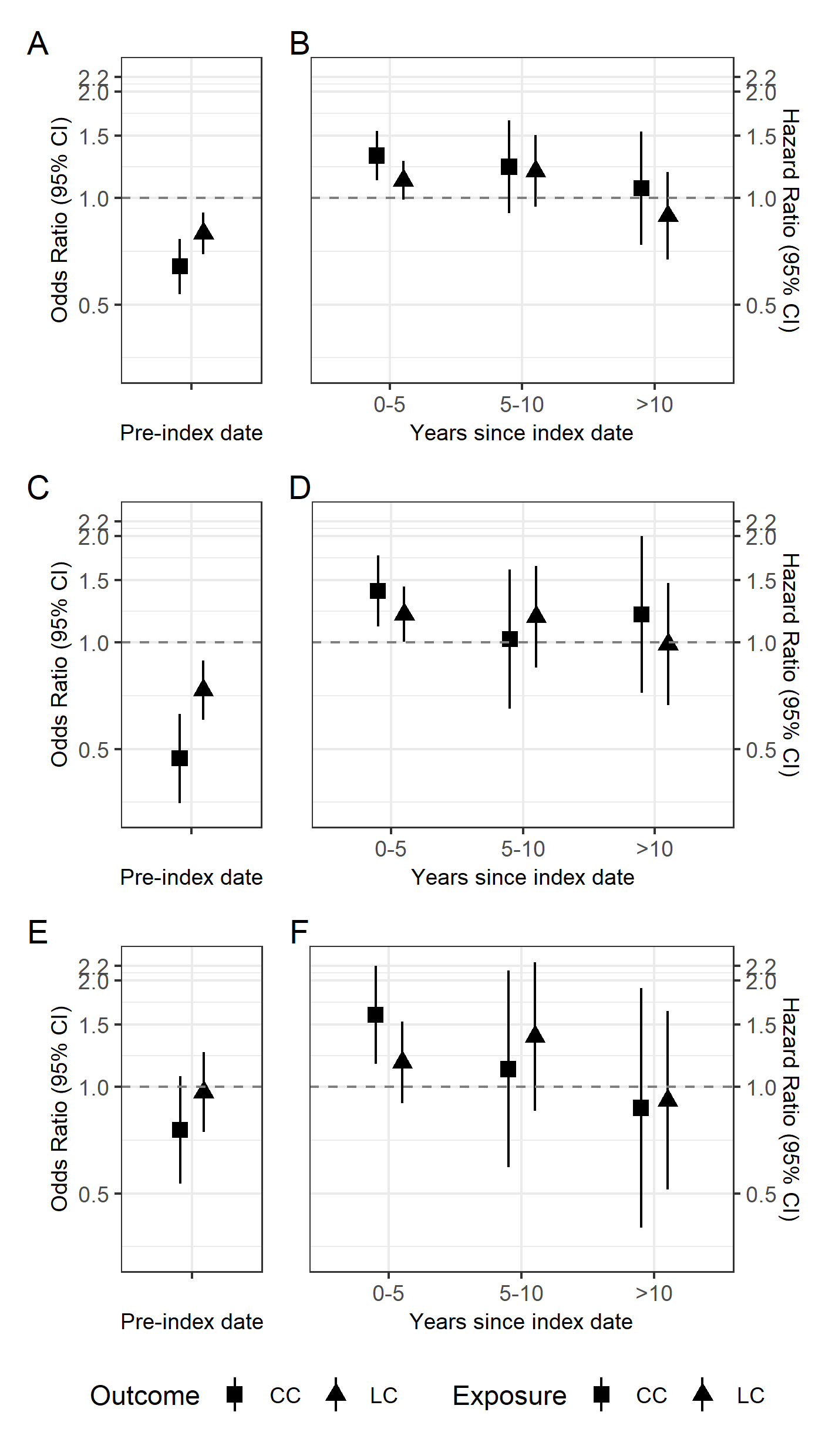

Supplement: Supplementary file 1 — Table S1: The Swedish‐version International Classification of Diseases (ICD) and Anatomical Therapeutic Chemical (ATC) codes used in the present study. Table S2: Baseline characteristics of samples included in the matched case‐control analyses. Table S3: Medication‐adjusted association of microscopic colitis with prevalent dementia among the sub‐sample with index date between 2006 and 2017. Table S4: Associations of inflammatory bowel disease with prevalent dementia. Table S5: Bidirectional associations of collagenous colitis and lymphocytic colitis, respectively, with dementia and its subtypes. Fig. S1: Cumulative incidence of dementia and its subtypes among patients with microscopic colitis and their comparators. Fig. S2: Adjusted hazard ratios for MC association with dementia and its subtypes estimated from flexible parametric models. Fig. S3: Bidirectional associations between collagenous colitis and lymphocytic colitis, respectively, and dementia by subtype. [file JOIM-299-216-s001.docx]
